# Synthesis and characterization of non-viral liposomal carriers for the local application of siRNA molecules and anti-miRNAs in the therapeutic treatment of psoriasis

**DOI:** 10.1186/1479-5876-9-S2-P13

**Published:** 2011-11-23

**Authors:** Stefanie Bracke, Barbara Geusens, Peter Dynoodt, Mireille Van Gele, Reinhart Speeckaert, Joost Schalkwijk, Sandra Tjabringa, Jo Lambert

**Affiliations:** 1 Dept. of Dermatology, Ghent University Hospital, Ghent, Belgium; 2 Dept. of Dermatology, Radboud University Nijmegen Medical Centre, Nijmegen, The Netherlands

## Background

Psoriasis is a common inflammatory skin disease with a multifactorial genetic basis. A dysregulated interplay between keratinocytes and infiltrating immune cells underlies the cutaneous inflammation in psoriasis. Keratinocytes are important producers of antimicrobial peptides such as hBD-2 and LL37 and cytokines such as TNF-alpha, which are essential elements in this process of cell-cell communication [1]. Recently, miRNA-203 was identified as an important contributor to this dysfunctional cross talk [2]. We have previously developed a new lipid-based nanosome (SECosome) that enables the effective delivery of siRNA into human skin [3]. The aim of this project is to knockdown mRNA encoding hBD-2, LL37, TNF-alpha and miRNA-203 by tranfection of keratinocytes with SECosomes for the delivery of siRNAs and anti-miRNAs. Ultimately, we want to create a new therapy for psoriasis by intervening at genetic level by means of a topical therapy.

## Materials and Methods

An optimized cytokine mix was used to induce a psoriatic phenotype starting from normal human keratinocytes. Complexes of siRNA or anti-miRNA and SECosomes were made and validated prior to transfection. 24h post-tranfection, qPCR analysis was performed to evaluate mRNA expression levels.

## Results

Transfection experiments with the complexes showed a stable knockdown efficiency of more than 80% of hBD-2, LL37, TNF-alpha and miR-203 mRNA.

## Conclusion

In this *in vitro* work we prepared and characterized siRNA and anti-miRNA complexes with SECosomes against hBD-2, LL37, TNF-alpha and miR-203 respectively. These complexes efficiently knock-down the targeted genes with concomitant downregulation of the associated proteins. Hereafter we will test the therapeutic applicability of our complexes in xenografted psoriatic skin by topical application.
